# Comparison of Automated Ribotyping, *spa* Typing, and MLST in 108 Clinical Isolates of *Staphylococcus aureus* from Orthopedic Infections

**DOI:** 10.3390/ijms23031660

**Published:** 2022-01-31

**Authors:** Stefano Ravaioli, Davide Campoccia, Werner Ruppitsch, Franz Allerberger, Alessandro Poggi, Emanuele Chisari, Lucio Montanaro, Carla Renata Arciola

**Affiliations:** 1 Laboratorio di Patologia delle Infezioni Associate all’Impianto, IRCCS Istituto Ortopedico Rizzoli, via di Barbiano 1/10, 40136 Bologna, Italy; stefano.ravaioli@ior.it (S.R.); lucio.montanaro@unibo.it (L.M.); 2 Division of Human Medicine, Austrian Agency for Health and Food Safety, 1010 Vienna, Austria; werner.ruppitsch@ages.at (W.R.); franz.allerberger1@gmail.com (F.A.); 3 Department of Biomedical, Metabolic and Neural Sciences, Section of Public Health, University of Modena and Reggio Emilia, 41125 Modena, Italy; alle.poggi90@gmail.it; 4 Rothman Orthopaedic Institute, Thomas Jefferson University, Philadelphia, PA 19107, USA; emanuele.chisari@rothmanortho.com; 5 Department of Experimental, Diagnostic and Specialty Medicine, University of Bologna, via San Giacomo 14, 40126 Bologna, Italy; 6 Laboratorio di Immunoreumatologia e Rigenerazione Tissutale, IRCCS Istituto Ortopedico Rizzoli, via di Barbiano 1/10, 40136 Bologna, Italy

**Keywords:** *Staphylococcus aureus*, orthopedic infections, strain typing, Riboprinting, multilocus sequence typing (MLST), *spa* typing

## Abstract

108 isolates of *Staphylococcus aureus*, belonging to six large ribogroups according to the automated Ribo-Printer^®^ system, were studied with two highly used molecular methods for epidemiological studies, namely multi-locus sequence typing (MLST) and *spa* typing, followed by BURP and eBURST v3 analysis for clustering *spa* types and sequence (ST) types. The aim was to evaluate whether automated ribotyping could be considered a useful screening tool for identifying *S. aureus* genetic lineages with respect to *spa* typing and MLST. Clarifying the relationship of riboprinting with these typing methods and establishing whether ribogroups fit single clonal complexes were two main objectives. Further information on the genetic profile of the isolates was obtained from *agr* typing and the search for the *mecA*, *tst* genes, and the IS*256* insertion sequence. Automated ribotyping has been shown to predict *spa* clonal complexes and MLST clonal complexes. The high cost and lower discriminatory power of automated ribotyping compared to *spa* and MSLT typing could be an obstacle to fine genotyping analyzes, especially when high discriminatory power is required. On the other hand, numerous advantages such as automation, ease and speed of execution, stability, typeability and reproducibility make ribotyping a reliable method to be juxtaposed to gold standard methods.

## 1. Introduction

*Staphylococcus aureus* is a leading pathogen in infections associated with orthopedic implants [1,2,3,4]. Originally described using multi locus enzyme electrophoresis (MLEE) and pulsed field gel electrophoresis (PFGE), and subsequently confirmed by multi-locus sequence typing (MLST) [5], the highly clonal structure of *S. aureus* population underlines the flexibility of *S. aureus* to evolve into an assortment of different clones.

Sequence-based typing methods, such as MLST and *spa* typing, characterized by a universal and worldwide nomenclature and by organized databases and networks (e.g., http://spaserver.ridom.de, http://www.seqnet.org/, http://saureus.beta.mlst.net/; accessed on 26 January 2022), are becoming the gold standards for epidemiological and for microbial population genetic investigations. In fact, the increase of variations by mutation occurs approximately linearly with time, and the resulting genetic distance tends to be proportional to the time of divergence between alleles, particularly in MLST [6].

At present, MLST is the most diffuse and highly discriminative typing method, based on the sequencing of seven housekeeping genes. The alleles at these seven loci are compared to an online database for each gene and provide an allelic profile represented by a distinct numerical identifier. A given allele’s combination generates an allelic profile which defines the sequence type (ST) that unequivocally identifies a clone. By this technique, linked STs that share 6/7 alleles (single locus variants, SLV) or at least 5/7 alleles (double locus variants, DLV) may be clustered together by eBURST software (http://eburst.mlst.net/; accessed on 26 January 2022) into genetically related groups referred to as ‘clonal complexes’ (CC), useful to assess the population organization and the patterns of evolution [7].

*Spa* typing is another commonly used sequence-based typing method for *S. aureus* isolates, which targets the polymorphic variable number tandem repeat (VNTR) region of the staphylococcal protein A gene (*spa*). The *spa* repeats are in general 24 bp in length, and with their putative mutations, duplications, deletions, and rearrangements, contribute to the identification of more than 15,100 distinctive patterns identified as ‘*spa* types’. Relatedness between *spa* types is analyzed by Based Upon Repeat Patterns (BURP) algorithm. *spa* CCs obtained by BURP analysis generally display an excellent concordance with MLST CCs [8]. As for MLST, *spa* typing data is unambiguous, portable and has a database accessibility, which permits intra- and inter-laboratory comparability. Moreover, this technique is cost-effective, involves easy processing, allows a rapid throughput of samples, and is dynamically evolving [9].

Ribotyping is a classic variant of the southern hybridisation-mediated assay that assesses the number of ribosomal gene loci and their collocation in the genome [10,11]. Bacterial ribosomes contain three distinct types of rRNA, the 16S, the 23S, and the 5S rRNA. Their encoding genes are orderly organized into the operon (*rrn*), which also includes a spacer region between the genes encoding for 16S and 23S rRNAs. The genetic peculiarity of 16S rRNA gene makes it one of suitable tools among phylogenetic markers for the evolutionary relationships between bacteria [12]. More than a copy of the *rrn* operon can often be found within a single bacterial genome. While the rRNA genes are at least 99.5% identical among multiple copies of the operon of the same microorganism, the spacer regions, including their internally transcribed spacer (ITS) region, can substantially differ in sequence and structure [12].

Automated ribotyping (riboprinting), which is performed using the RiboPrinter^®^ microbial characterization system (Qualicon), consists of the analysis of the genomic fragments generated by the enzymatic restriction of the rRNA operon, frequently found in multiple copies (from 1 to 15) in the bacterial genome [13]. Whole genome sequencing analysis has shown as the number of copies of the *rrn* operon varies not only across species of the same genus, but also within each single species. For instance, in *S. aureus* the number of copies of *rrn* operons was found to vary between four and six (http://rrndb.umms.med.umich.edu/; accessed on 26 January 2022), with six copies being the conditions most frequently observed. As aforementioned, the DNA regions encoding ribosomal RNA are highly conserved, but the flanking sequences result in variations that are significant for the diversities of the ribopatterns [14]. The genetic peculiarity of 16S gene makes it one of suitable tools among phylogenetic markers for the evolutionary relationships between bacteria [12]. The restriction enzymes selected by Stoddard et al. [12] for the digestion of chromosomal DNA were *Eco*RI and *Hid*III. However, *Eco*RI was the best enzyme used, since, producing more bands and ribotypes than *Hid*III within the same *Staphylococcus* species, it can discriminate between more genetic types [15]. The number of bands distinguished by manual or automated ribotyping depends on the quantity of recognitions sites of the restriction enzyme and on the polymorphisms that are present within the *rrn* operons and their flanking regions.

Automated ribotyping is easy to execute and, due to the complete automation and standardisation, the riboprinting patterns have successful typeability, high reproducibility, and stability. In fact, the patterns of repeated isolates were always the same also after one year period [11,15,16]. Moreover, the reliability and rapidity (approximately 2–3-fold greater than manual ribotyping) make this method an ideal system in the clinical laboratory when identification rather than characterization is requested [14].

This study aimed at analysing by *spa* typing and by MLST 108 *S. aureus* isolates belonging to six major ribogroups present in our database, identified by the RiboPrinter^®^ microbial characterization system. Moreover, the study aimed at understanding the relation between the most widely used typing techniques and riboprinting, and at detecting the correspondence of ribogroups with MLST and *spa* CC. Additional information on the genetic profile of the isolates belonging to the same clonal complexes was obtained from *agr* typing and from the search for the *mecA*, *tst* and IS*256* genes.

## 2. Results

### 2.1. Results of the Automated Ribotyping Analysis

The outcomes resulting from the comparative analysis of the three distinct typing genotypic methods, respectively ribotyping, *spa* typing, and MLST, on the 108 clinical isolates of *S. aureus* are shown in Table 1 and Table 3. Automated ribotyping identified nine different ribotyping patterns. However, three of them were merged in a unique ribocluster, based on their high similarity, as reported in a previous work [17]. In fact, the three ribogroups *cra*-119-S8, *cra*-138-S-2 and *cra*-53-S-7, constituted respectively by 17, six, and four isolates, were merged in a larger one of 27 isolates hence called ribocluster. In the present study, that ribocluster has been further extended to include 21 additional isolates, thus obtaining a ribocluster consisting of 48 strains. The other ribogroups in order of frequency were *cra*-94-S-7 (16 isolates), *cra*-137-S-4 (14 isolates), *cra*-157-S-4 (13 isolates), *cra*-146-S-8 (9 isolates), and *cra*-147-S-6 (8 isolates), as reported in Table 1.

### 2.2. Results of the MLST Analysis

The MLST analysis generated 19 STs, these including five new, previously unreported, STs, plus an additional non-typeable ST (Table 1). The isolates belonged to five clonal complexes, namely CC30, CC8, CC7, CC5, and CC1 and a sporadic CC, CC45. The population structure analysis based on eBURST v3 algorithm with the limit of 5 SLV produced 4 groups and singletons, namely ST1, ST109, ST101, and ST46.

### 2.3. Relatedness between Ribogroups, STs, MLST CCs and eBURST Groups

As reported in Table 1, the majority of the 48 strains of the ribocluster belonged to two MLST strain types: ST30 (64.6%) and ST34 (20.8%). The other less represented strains belonged to: ST243, ST2954, ST2957, and ST2960. A further strain resulted a non-typeable ST. All of them formed part of the CC30 (this was perfectly aligned with our previously published findings [17]) and of Group 1 founded by eBURST v3 analysis (DLV limit).

The ribogroup *cra*-94-S-7 consisted prevalently of strains identified as ST228 (87.5%). The 14 strains ST228 and an additional strain belonging to a previously unknown ST, namely ST2956, were all part of the CC5 and of the eBURST Group 2. A further strain ST46 was attributed to CC45 and to the ST46 singleton, as per eBURST identification. A similar ribogroup, *cra*-146-S-8, consisted uniquely of strains belonging to CC5 and eBURST Group 2. In this case, ST5 represented the most frequent ST (87.5%).

All 14 strains of the ribogroup *cra*-137-S-4 belonged to CC8 and eBURST Group 3, with ST8 (64.3%) and ST247 (28.6%) being the two prevailing STs and ST241 being represented by a single strain. The ribogroup *cra*-157-S-4 saw the prevalence of strains ST7 (69.2%) and included one strain, ST2955, a new, previously unreported, strain type. Although most of its strains belonged to CC7, a pair of strains were ST109 and associated to CC1 and one strain was ST101, for which the CC was not delivered. The strains of CC7 were clustered by eBURST in Group 4, while both ST109 and ST101 were classified as singletons. The ribogroup *cra*-147-S-6 consisted uniquely of strains ST1 (CC1), which were grouped in the ST1 singleton by eBURST analysis.

Overall, ribogroups *cra*-94-S-7 and *cra*-146-S-8 were found to be associated to CC5, the ribocluster to CC30, the ribogroup *cra*-137-S-4 to CC8 and the ribogroup *cra*-147-S-6 to CC1. Based on the above data, the ribogroups of the isolates would predict the MLST CC in a proportion of strains ranging from 76.9 to 100%, which is a remarkable result giving the different methodological approach adopted for strain typing.

### 2.4. Relatedness between Ribogroups, spa Types, spa CCs and BURP Clusters

**Figure 1:**
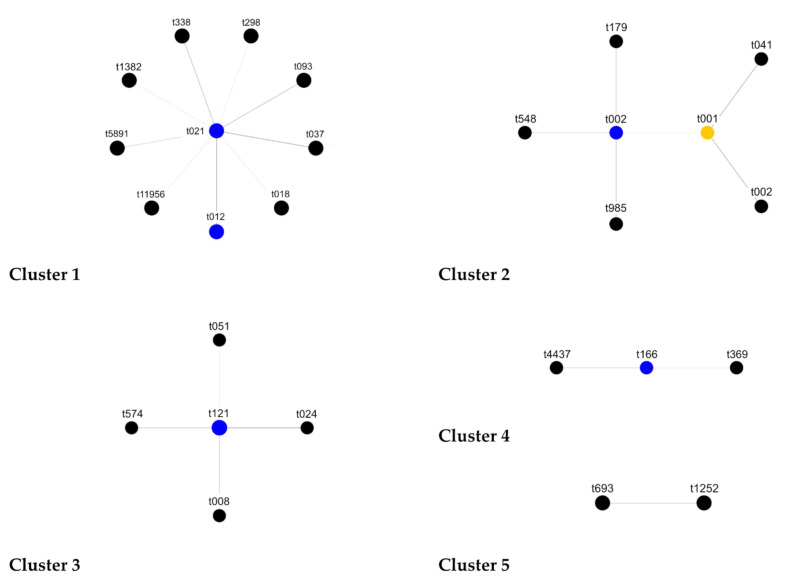
Clusters founded by BURP software analysis.

The characterization by *spa* typing discriminated 39 *spa* types, while the BURP analysis clustered 82 isolates in four *spa* clonal complexes (*spa* CC), namely *spa* CC021/012, *spa* CC166, *spa* CC002 and *spa* CC121, and classified 24 isolates as singletons (Table 2 and Figure 1). Two sporadic isolates (t693 and t1252) were excluded, having the sequences shorter than 5 repetitions. Among the 39 different *spa* types, three *spa* types (t13129, t13130 and t13132) were discovered as totally new.

In total, three isolates with *spa* type t1382 belonging to *spa* CC021/012, were observed in two different ribogroups, respectively in the ribocluster (2 isolates) and in *cra*-94-S-7 ribogroup (1 isolate) (Table 2).

The clonal complex *spa* CC021/012, prevalently correlated with the ribocluster, was the most frequent *spa* CC among all the 108 isolates (34%), followed by *spa* CC121 (12%), *spa* CC166 (7%), and *spa* CC002 (6%). Moreover, two ribogroups were not associated with any specific *spa* CC, namely: *cra*-157-S-4, which was structured by only singletons, and *cra*-147-S-6, which was structured by singletons and along with the two excluded isolates (Table 2).

Differently from what was observed with MLST CCs, ribotyping could less effectively predict *spa* CCs and *spa* clusters. For instance, in *cra*-147-S-6, only 25% of the strains belonged to Cluster 5 and none to a perfectly identified CC. Similarly, *cra*-157-S-4 included only singletons and none of the strains was attributed to a spa CC or to a cluster. *cra*-146-S-8 and *cra*-94-S-7 were composed for 66.7% and 87.5% of strains CC002 (Cluster 2), respectively. 77.1% of the strains enlisted in the ribocluster were CC021/012 (Cluster 1). An exception was observed with *cra*-137-S-4 consisting of 92.9% of strains CC121 and Cluster 3, and showing up to 87.5% of the strains belonging to CC002(Cluster 2). Thus, ribotyping never reached 100% ability to predict *spa* CCs. Conversely, MSLT CC could be predicted by ribotyping in 100% of the strains of the ribocluster and other 3 ribogroups: *cra*-146-S-8, *cra*-137-S-4, and *cra*-147-S-6. Further considerations in this regard may be made on the strange segregation of CC021/012 (Cluster 1), which, although prevailing within the ribocluster, was found to segregate into two further ribogroups: *cra*-94-S-7 and *cra*-137-S-4. Such misalignment occurred even with the MLST CC identifications as CC021/012 strains were observed to belong to MLST CC30, CC8, and CC5, suggesting that ribotyping and MLST shared greater similarity when predicting the relatedness of the isolates of *S. aureus* with respect to *spa* typing. Given the different methodological approaches existing between the three methods, whose two based on gene sequencing and one based on Southern blotting and restriction fragment length polymorphism (RFLP), it is at least in part surprising that MLST results at CC level seem more consistent with ribotyping than with *spa* typing.

### 2.5. Ribogroups Association with agr Type, tst and mecA Genes, IS256 and Phenotypic Antibiotic Resistance

Table 3 shows a high correlation between *agr* types and the prevalence of *mecA*, IS*256* and *tst* genes, within respective ribogroups, confirming the results of previous works. Moreover, the phenotypic antibiotic resistance traits followed the same patterns between isolates belonging to the same ribogroup.

As reported in Table 3, most of the strains of the large ribocluster exhibited an *agr* type III (93%), were endowed with the *tst* (83%) gene, resistant to the antibiotics PEN and AMP, but lacking IS*256*. All strains were negative to the *mecA* gene, thus largely susceptible to OXA. These findings confirm our previous observations [17]. Conversely, the two ribogroups *cra*-94-S7 and *cra*-146-S8, whose strains mostly belonged to CC5, exhibited very distinct genotypic and phenotypic traits except for the *agr* type II, which was highly prevalent (respectively in 94% and 100% of the strains), the *tst* gene (6% and 22%), and antibiotic resistance to three antibiotics, VAN, SXT, and CHL. Indeed, *cra*-94-S7 showed a high frequency of strains endowed with IS*256* (94%), *mecA* gene (88%), and resistant to OXA, PEN, AMP, GEN, ERY, CLI and CIP. The high prevalence of its resistant/multiresistant strains was reflected by the MAR index [18,19] that reached its highest value of 0.66. On the other hand, *cra*-146-S8 exhibited a MAR of 0.11, which was the lowest observed across ribogroups. Even the generally frequent resistance to PEN and AMP concerned less than 50% of the strains. The ribogroup *cra*-137-S4 consisted uniquely of *agr* type I strain, lacking the *tst* gene, and resistant to both PEN and AMP. IS*256* was present just in half of its strains, while the *mecA* gene was detected in a much larger proportion (79%). The MAR reached a value of 0.57 reflecting a high prevalence of strains resistant to the different antibiotics assayed, in particular GEN, ERY, and CIP. Similarly, *cra*-157-S4 included prevalently *agr* type I strains, all lacking the *tst* and *mecA* genes and the insertion element IS*256*. Its MAR value was 0.18 and reflected a particularly low frequency of antibiotic resistant strains, apart from PEN and AMP. Finally, *cra*-147-S6 consisted of *agr* type III strains. A quarter of the strains was endowed with the *tst* and *mecA* genes, while IS*256* was detected in half of its strain population. The MAR (0.24) indicated an antibiotic resistance just slightly lower than the average MAR calculated on all 108 strains (0.31).

**Table 3:** Overall prevalence of *agr* type, IS*256, tst,* and *mecA* genes, and phenotypic antibiotic resistance traits within each ribogroup.

Ribogroup	*Prev.* *agr* type	*tst*	IS*256*	*mecA*	OXA	PEN	AMP	GEN	ERY	CLI	CHL	SXT	CIP	VAN	MAR
Ribocluster	III 98%	83%	0%	0%	2%	**94%**	**92%**	4%	11%	6%	0%	0%	4%	0%	0.21
*cra*-94-S7	II 94%	6%	94%	88%	88%	94%	94%	94%	94%	94%	6%	0%	94%	0%	0.66
*cra*-146-S8	II **100%**	22%	0%	22%	0%	44%	44%	0%	0%	0%	0%	0%	0%	0%	0.11
*cra*-137-S4	I **100%**	0%	50%	**79%**	**86%**	**100%**	**100%**	**57%**	**57%**	**62%**	21%	7%	**86%**	0%	0.57
*cra*-157-S4	I 85%	0%	0%	0%	0%	**77%**	**77%**	8%	8%	8%	8%	0%	0%	0%	0.18
*cra*-147-S6	III **100%**	25%	50%	25%	25%	**75%**	**75%**	13%	25%	25%	0%	0%	0%	0%	0.24

Percent frequency of strains resistant to each antibiotic, with the insertion sequence IS*256*, and endowed with the genes tst and *mecA*. The average MAR value for all the 108 strains and all 10 antibiotics was 0.315. Legend: oxacillin (OXA); penicillin (PEN); ampicillin (AMP); gentamicin (GEN); erythromycin (ERY); clindamycin (CLI); chloramphenicol (CHL); trimethoprim-sulfamethoxazole (SXT); ciprofloxacin (CIP); vancomycin (VAN).

### 2.6. Uncommon STs and spa Types Atypically Found within Ribogroups

Table 4 reports the genetic characteristics of some uncommon STs and *spa* types that have been identified within three ribogroups and appear atypical, as they markedly differ from the other more prevalent STs, and *spa* types observed. ST46, detected only once, shows a sequence type different from all the others of the ribogroup *cra*-94-S-7 constituted by 16 isolates. In fact, it differed by all six alleles with respect to the most frequent ST228, and its t1646 *spa* type was very dissimilar from both t001 and t041. BURP analysis generated the singleton t1646 *spa* type, whereas the ribogroup was constituted by *spa* CC002.

Other atypical STs were identified in the *cra*-157-S-4 ribogroup, i.e., ST109 and ST101, which differed from the prevalent strains ST7 by five and six alleles, respectively. Their *spa* types (t209 and t7956 respectively) were also very dissimilar from the principal t091 *spa* type of that ribogroup.

Some strains such as *cra*2198 with the genetic profile ST241/t037 (Table 2) exhibited ambiguous connotations. In terms of allele succession, the sequence type ST241 showed similarity, with the genetic founder ST8, but its *spa* type t037 clustered into *spa* CC021/012 (Cluster 1).

### 2.7. Hunter-Gaston Discriminatory Power Analysis

The Hunter-Gaston discriminatory power of *spa* typing, ribotyping, and MLST obtained on the 108 *S. aureus* is shown in Table 5. For all 108 *S. aureus*, the method with the highest discriminatory index was *spa* typing (D = 0.955), followed by MLST (D = 0.869), and ribotyping (D = 0.744). Ribotyping discriminatory index indicates a low or moderate ability to create different genotypes. Moreover, considering MSSA isolates, the discriminatory index was superior to MRSA considering both *spa* typing and MLST. In fact, for *spa* types, the discriminatory power was of 0.936 vs. 0.879, while for MLST ST was of 0.802 vs. 0.751, and considering the *spa* CC, the discriminatory power was of 0.746 vs 0.594 while for MLST CC was of 0.590 vs 0.567. Table 6 and Table 7 illustrate the genotypic characteristics of MSSA and MRSA strains (*spa* types and CC, MLST ST and CC) inside the ribogroups. The data obtained clearly confirm the prevalence between MSSA strains of more genetic lineages respect MRSA and then, excluding ribotyping, a major discriminatory power.

## 3. Discussion

In the present study, 108 *S. aureus* isolates belonging to the six larger ribogroups, determined by automated RiboPrinter^®^ System, were investigated by the two molecular methods most used for epidemiological studies, i.e., MLST and *spa* typing, followed by BURP and eBURST v3 analysis to cluster the *spa* types and STs (Sequence Types) which originated. The strain variety, the discriminatory power, and the typeability were used to assess the efficiency of the genotyping methods.

### 3.1. MLST vs. Automated Ribotyping

Relationships between ribogroups and STs were shown in Table 1. There was a good concordance between the genetic lineages created by MLST and automated ribotyping. MLST evidenced 19 STs, five of them being new STs (ST2954, ST2957, ST2960, ST2956, and ST2955), plus 1 non-typeable isolate. Each ST was characteristic of a specific ribogroup since it was not found in other ribogroups. Ribotyping assigned strains with specific STs as ST30 (64.6%) with to the riboclusters (*cra*-119-S-8, *cra*-138-S-2, and *cra*-53-S-7 ribogroups), ST228 (87.5%) to *cra*-94-S-7 ribogroup, ST8 (64.3%) to *cra*-137-S-4 ribogroup, ST7 (69.2%) to *cra*-157-S-4 ribogroup, ST5 (88.9%) to *cra*-146-S-8 ribogroup and ST1 (100%) to *cra*-147-S-6 ribogroup (Table 1).

MLST and ribotyping, referring to highly conserved nature of the housekeeping genes and *rrn* operon examined, could be considered good tools for long-term and large-scale evolution of a vast spectrum of microorganism, being the clock speed of their DNA alterations moderately slow.

Comparative ribotyping and MLST-based dendrograms for *H. influenzae* and *S. pneumoniae*, constructed with large sets of about 500 isolates each, proved to be highly congruent [14].

A fine relation between MLST and automated ribotyping was previously shown by McAleese et al. [20] in a group of CA-MRSA strains, coming from skin and intra-abdominal infections of patients in part from USA and from Europe. In that work, ST8 and ST1 were strictly linked respectively to the A2 and G2 ribotypes. In that study, the use of ribotyping was considered a fine tool to group MRSA with similar genetic background. As previously seen, and also in this study, *cra*-94-S-7, and *cra*-137-S-4 ribogroups, constituted for the mostly part by MRSA strains, had particular STs, as ST228 (87,5%) and ST8 (64,3%) (Table 1). Moreover, the other STs assigned to the same ribogroup were correlated and had similar allele succession. On the contrary, four isolates, namely *cra*1143 (ST46-t1646), *cra*2578, *cra*3029 (ST109-t209), and *cra*3164 (ST101-t7956), had atypical STs compared to the ones of the other isolates assigned to the same ribogroup (Table 1 and Table 4).

Although automated ribotyping is considered a reliable method for identifying different bacterial species, easy to perform, with a high reproducibility, high typeability, and stability, its relatively limited discriminatory power is an obstacle to be used as a reference technique in genotyping method [11]. rRNA 16S, 23S, and 5S are encoded by the polycistronic *rrn* operon which is present in different copies with a range between one (*Rickettsia prowazekii* and *Mycoplasma pneumoniae*) and 15 (*Clostridium paradoxum*), depending on the bacterial species [21]. *Staphylococcus aureus* exposes the presence of a range between four and six copies of *rrn* operons (http://rrndb.umms.med.umich.edu/; accessed on 26 January 2022); then, considering that rRNA operons number commonly raises the discriminatory power, a moderate discriminatory power is obtained.

MLST method is considered as the current gold standard method for microbial population dynamics and global genetic lineages analysis and quickly has become the gold standard for epidemiological surveillance. MLST is useful than ribotyping to relate inter-laboratory results with a standardized taxonomy having an online database increased over the time, making possible the sharing and investigation of MLST data [11].

### 3.2. spa Typing vs. Automated Ribotyping

*spa* typing is recommended as a fine genotyping method for a national and international epidemiological control as well as for short time and local surveillance [22].

As shown in Table 2, *spa* typing of the 108 *S. aureus* strains gave 39 *spa* types and three of these were new types observed for the first time in this study, namely, t13129, t13130, and t13132. Analyzing the relationship between *spa* typing and ribogroups, each *spa* type is generally characteristic for a specific ribogroup.

A significant incongruence was found, namely t1382, belonging to *spa* CC021/012, was observed in three strains assigned to two different ribogroups and to two different MLST CCs. In fact, two of these strains with the genetic profile t1382-ST30 belong to the ribocluster-MLST CC30, mainly constituted by strains assigned to *spa* CC021/012 (37 isolates out of 48), while the other strain had the different genetic profile t1382-ST228 and was assigned to *cra*-94-S-7 ribogroup-MLST CC5, mainly constituted by strains assigned to *spa* CC002 (14 isolates out of 16).

This finding should not be considered eccentric, since “*spa* type” genotypes are based on highly variable X region sequences of the gene encoding for surface protein A, and therefore it is not obvious that this gene match with the same evolutionary lineages observed using the other molecular epidemiologic methods as ribotyping and MLST. Moreover, a confirmation of this was observed in the isolate carrying the *spa* type t037 clustered into *spa* CC021/012 and assigned to a ribogroup (namely *cra*-137-S-4), whereas all of the other strains had dissimilar *spa* repeat succession and were clustered into *spa* CC121 (Table 2 and Table 4). In this case, its MLST sequence type (ST241) showed a similarity, in term of allele succession, with the genetic founder ST8, clustered into MLST CC8. This observation validates that the evolutionary histories of isolates, (taking in consideration *spa* typing and MLST methods) are not always linked and the mutations on the seven housekeeping alleles and that *spa* repeat succession cannot always be predicted and do not follow any precise scheme.

Furthermore, as previously evidenced, four strains had with unusual STs had in addition atypical *spa* types respect the other strains assigned to the same ribogroups (Table 4).

Considering *spa* typing and MLST, more genetic assortments were detected in MSSA isolates. The discriminatory power of *spa* typing and MLST for MSSA isolates was 0.936 and 0.802, respectively (32 *spa* types and 16 STs), while for MRSA isolates, the discriminatory power was of 0.879 and 0.751, respectively (11 *spa* types and 7 STs). This finding was similar to those observed in previous studies [23,24,25,26]. In fact, the genetic lineage population of MSSA has in general a diversity in genetic assortment greater than MRSA lineages [22,26,27,28,29,30]. In some instances, there were not specific MLST STs and *spa* types for discriminating among MSSA and MRSA strains (Table 6A,B).

### 3.3. Clonal Complexes

As observed from the results shown in Table 1, automated ribotyping and eBURST v3 analysis had higher concordant results between ribotyping and BURP analysis, since ribogroups corresponded to determine groups and MLST clonal complexes, excluding the CC5 (group 2 from eBURST analysis), which was represented by two different ribogroups (*cra*-94-S-7 and *cra*-146-S8) (Table 1 and Table 2). The ribocluster matched with CC30 (group 1), *cra*-137-S-4 ribogroup with CC8 (group 3), *cra*-147-S-6 ribogroup with isolates corresponded to CC1 (singletons), and *cra*-157-S-4 ribogroup corresponded to CC7 (group 4 and three singletons).

BURP analysis of *spa* types generated numerous singletons. The *cra*-157-S-4 and *cra*-147-S-6 ribogroups were constituted by only singletons. As for eBURST v3 analysis, where Group 2 (MLST CC5) corresponded to *cra*-94-S-7 and *cra*-146-S-8 ribogroups; additionally, BURP analysis created a single *spa* CC002 from the strains assigned to two *cra*-94-S-7 and *cra*-146-S-8 ribogroups. On the contrary, two *spa* CCs (*spa* CC021/012 and *spa* CC166) were merged in the same ribocluster and MLST CC30.

The outcome was that BURP and eBURST v3 analysis were more linked to each other than to with ribotyping.

*Spa* CC021/012 is found in three ribogroups, i.e., the ribocluster, the *cra*-94-S-7, and the *cra*-137-S-4 ones ribogroups, even if, as previously observed, there were only two isolates with *spa* types t1382 and t037, belonging to the *cra*-94-S-7 and the *cra*-137-S-4 ribogroups (Table 2 and Table 6). This finding can be explained considering the different nature of genes analyzed with the different genotyping methods, which could group in the same cluster by one method, even if strains were clustered into the same clone by another method.

Therefore, there is a better accordance between MLST CCs and ribogroups than between *spa* CCs and ribogroups, and it could be explained by the intrinsic nature of *spa* typing which is a single-locus sequence typing (SLST) pertaining to the *S. aureus* protein A gene, whose repeats are moderately mutable and variable in number, while for DNA sequences, analyzed with MLST and automated riboprinting, genetic variations were slower than those for protein gene A, since polymorphism evolves gradually in *rrn* operon and housekeeping genes [11].

While *spa* typing and MLST are considered gold standard and have a high discriminatory power, the combination of the three typing methods are more instructive tools for epidemiological studies on *S. aureus* strains and useful to perform a clearer evaluation of the outbreaks. It has been suggested that the use of two or more genotyping methods may attain a better estimation of the genetic diversities of isolates [31].

Automated ribotyping, with respect to the other typing methods, was unable to discern difference between the groups of isolates of this study, then had the ability to group strains with different genetic pattern in congruence with MLST and *spa* typing.

The discriminatory power of the genotyping methods is the crucial argument to take into consideration in the context of a predominant spreading clone [31]. Despite the disadvantage that automated ribotyping may not be the method for genotyping when discriminatory power is needed, it offers the possibility to correlate clone complexes with virulence factors, i.e., adhesins, toxins, antibiotic resistances, capacity to form biofilm (Table 3), etc. Automated ribotyping could be useful to identify isolates belonging to virulent clones, with particular interest to phylogenetic studies between members of the same taxon [17,32,33].

Ribotyping could therefore be an advantage in a preliminary study where it is required clinical isolates that possess distinct pathogenic characteristics (Table 3). The study of Blasi F et al. [34] exhibits that *C. difficile* strains belonging to the same ribotypes have the same toxinotypes and antimicrobial resistance profiles. Additionally, it would be an additional value to improve the information obtained from *spa* typing and MLST in order to associate the identified clones with probable emerging global clones with special genetic lineages and also to verify the incidence of the same clones in the laboratory databases and to compare their genetic and phenotypic properties.

## 4. Conclusions

The goal of the current work is to evaluate if automated ribotyping could be considered as a helping screening tool to identify and differentiate *S. aureus* genetic lineages in relation with other golden methods as *spa* typing and MLST. For the multitude of *spa* types generated, *spa* typing, unlike ribotyping and MLST, proves a strong tool in an outbreak location, since endemic clones request high levels of discrimination.

The automated ribotyping, can predict in some cases the *spa* clonal complexes and, in a more balanced way, MLST clonal complexes. The high cost, similar to the MLST method one, and lower discriminatory power of automated ribotyping than *spa* typing and MSLT, could be an obstacle for genotyping analysis especially when high discriminatory power is needed. Otherwise, different advantages as automation, easy to perform, stability, typeability, rapidity (less than 8 h), and reproducibility make ribotyping a reliable method to juxtapose to the gold standard ones [11,33]. Combination of genotyping technique should be better and preferable over single technique if high discriminatory method is required [32]. *Spa* typing and MLST offer a multitude of genetic lineages which would otherwise have been overlooked by automated ribotyping; both of the methods use a universal nomenclature, which makes them helpful for collaborative networks.

Finally, automated ribotyping, indicating a relatedness among isolates with different genetic background, could be better for small or local analysis and regional or national investigations, instead of studies among internationally collaborative networks and explorations of worldwide disseminated clones and outbreaks.

## 5. Materials and Methods

### 5.1. Bacterial Isolates

A total of 108 *S. aureus* isolates, coming from revisions of surgical wounds and treatment of infected prostheses of patients coming from the entire Italian territory, were collected over a period of 13 years (2000–2012) and stored at −80 °C. The majority (82 samples, 76%) of the isolate was from orthopedic implant-related infections. The strains were deposited in the ISO 9001:2015 certified biobank of the Research Unit on Implant Infections at the Rizzoli Orthopaedic Institute (Bologna, Italy). The strains were identified by either or both Api-Staph and ID 32 Staph test (BioMérieux, Marcy l’Etoile, France) and confirmed by ribotyping analysis.

### 5.2. Antibiotic Susceptibility

The agar diffusion (Kirby-Bauer) method was utilized to perform the antibiotic susceptibility tests according to Clinical and Laboratory Standards Institute (CLSI) guidelines (NCCLS, 2002) [35]. Antimicrobial susceptibility was tested for a panel of 10 antibiotics: oxacillin (OXA), penicillin (PEN), ampicillin (AMP), gentamicin (GEN), erythromycin (ERY), clindamycin (CLI), chloramphenicol (CHL), trimethoprim–sulfamethoxazole (SXT), ciprofloxacin (CIP), and vancomycin (VAN).

### 5.3. Automated Ribotyping

To be subjected to ribotyping method, each strain was seeded on brain hearth infusion (BHI) agar plate for 24 h at 37 °C. A single colony of each isolate culture obtained on BHI agar plates was transferred to mannitol salt agar (MSA) plate specific for staphylococci growing. A pure colony from MSA agar plates of each isolate was processed by RiboPrinter^®^ microbial characterization system (Qualicon, Wilmington, DE, USA), following the manufacturer’s instructions. *Eco*RI was the restriction enzyme used for the analysis.

The identification of each isolate was obtained when its ribotype pattern matched the pattern of one of the reference isolates of the DuPont Identification Library with a similarity ≥ 0.85. Isolates with a pattern similarity ≥ 93% are clustered together in the same ribocluster. When the similarity with other previously analyzed isolates does not reach such a threshold, a new ribogroup is automatically generated. Conversely, the attribution of an isolate to a previously existing ribogroup automatically occurs when the similarity with the ribogroup average profile is ≥90%.

The RiboPrinter patterns were also imported and analyzed in BioNumerics version 4.6 (Applied Maths, Sint-Martens-Latem, Belgium). Pairwise similarities were calculated using the Pearson-product-moment correlation coefficient with 0.5% optimization and from the resulting similarity matrix a UPGMA (unweighted pair group method using arithmetic averages) dendrogram was derived. The Cluster Cut-off method of BioNumerics was utilized for the identification of the major clusters [36].

### 5.4. Bacterial DNA Isolation

The chromosomal DNA used as an amplification template was extracted from the bacterial cultures using QIAmp DNA mini kit (Qiagen, GmbH, Hilden, Germany), according to the manufacturer’s instruction, as previously described [37].

### 5.5. Detection of mecA, tst, IS256, and agr Typing

PCR conditions and primers of *mecA*, *tst* and IS*256* genes used in this study are previously described [38]. The identification of the *agr* polymorphisms was performed through PCR, as described by von Eiff et al. [39].

### 5.6. spa Typing

The polymorphic X, or short sequence repeat (SSR) region, of the *S. aureus* protein A gene (*spa*) was amplified with the forward (5′-TGTAAAACGACGGCCAGT-3′) and the reverse (5′-CAGGAAACAGCTATGACC-3′) primers according to the protocols previously defined [40,41]. A total of 10 microliters of the amplified products were analyzed on 1.5% agarose gels and 5 µL were purified with EXO SAP-IT (GE Health care, Buckinghamshire, UK). In total, two microliters of the purified amplification products were used for subsequent sequencing using the Big Dye Terminator v3.1 sequencing kit (Applied Biosystems, Carlsbad, CA, USA) and were finally analyzed on ABI Genetic Analyzer 3500 Dx (Applied Biosystems). The chromatograms obtained were analyzed with the Ridom StaphType software version 1.4 (Ridom GmbH, Würzburg, Germany; http://spa.ridom.de/index.shtml; accessed on 26 January 2022) to determine the *spa* type of each isolate [41]. *spa* types were deduced by the difference in number and sequence of *spa* repeats. For establishing the evolutionary relationship, *spa* types were clustered into different *spa* CCs and groups by using the BURP algorithm (Ridom GmbH) and the Ridom SpaServer database (http://www.spaserver.ridom.de; accessed on 26 January 2022).

### 5.7. MLST

MLST genotyping was performed on all 108 *S. aureus* isolates as described in [42]. The amplification of a portion of seven housekeeping genes (*arc*, *aroE*, *glp*, *gmk*, *pta*, *tpi*, and *yqiL*) was performed and successively sequenced. The sequences were analyzed using the bioinformatics software package UGENE 1.13 (Unipro) and the reference seven genes sequences were obtained on the MLST website (https://pubmlst.org/bigsdb?db=pubmlst_saureus_seqdef; accessed on 26 January 2022). Each allele was acquired by submitting the examined sequence to the *S. aureus* MLST database (https://pubmlst.org/bigsdb?db=pubmlst_saureus_seqdef&page=profiles&scheme_id=1; accessed on 26 January 2022), and finally the allelic profiles (sequence types, STs) and the CCs were allocated via the MLST database. With the eBURST v3 software (https://pubmlst.org/bigsdb?db=pubmlst_saureus_islates&page=plugin&name=BURST; accessed on 26 January 2022), sequence types (STs) were clustered to assign them to groups and to identify the potential ancestral type (AT). *S. aureus* isolates with STs differing for just one or two housekeeping genes/loci were assigned to a unique group, then a double locus variant (DLV) was the limit set for the analysis.

### 5.8. Discriminatory Power

MRSA and MSSA strains discriminatory power of ribotyping, *spa* typing, MLST, *spa* CC and MLST CC, was obtained using the Hunter-Gaston discriminatory index (HGDI; http://insilico.ehu.es/mini_tools/discriminatory_power/; accessed on 26 January 2022).

$$D=1-\frac{1}{N(N-1)}\sum_{J=1}^{S}n(nj-1)$$

where **N** is the total number of examined strains, **S** the total number of identified types and nj the total number of isolates belonging to the jth type.

## Figures and Tables

**Table 1:** Relatedness between ribogroups, STs, MLST CCs and groups founded by eBURST v3 analysis.

Ribogroup	ST (%)	MLST CC Founder (Breaker)	eBURST Groups **
Ribocluster (48)	**ST30** (64.6%) ST34 (20.8%) ST2954 * (6.2%) ST2957 * (2.1%) ST2960 * (2.1%) ST untypeable (2.1%) ST243 (2.1%)	CC30 (SLV)	Group 1
*cra*-94-S-7 (16)	**ST228** (87.5%) ST2956 * (6.25%) ST46 (6.25%)	CC5 (DLV) CC5 (DLV) CC45 † (SLV)	Group 2 Group 2 ST46 singleton
*cra*-146-S-8 (9)	**ST5** (88.9%) ST2626 (11.1%)	CC5 CC5 (SLV)	Group 2
*cra*-137-S-4 (14)	**ST8** (64.3%) ST247 (28.6%) ST241 (7.1%)	CC8 CC8 (DLV) CC8 (DLV)	Group 3
*cra*-157-S-4 (13)	**ST7** (69.2%) ST2955 * (7.7%) ST109 (15.4%) ST101 (7.7%)	CC7 CC7 (DLV) CC1 † (TLV) CC not delivered†	Group 4 Group 4 ST109 singleton ST101 singleton
*cra*-147-S-6 (8)	**ST1** (100%)	CC1	ST1 singleton

The CCs were evaluated with SLV and DLV (Single and Double Locus Variant). * New STs founded in this study and to be submitted to the MLST database; † atypical CCs found within the ribogroup. ** Groups founded by eBURST v3 analysis (DLV limit). The principal ST found for each ribogroup is reported in bold. STs in red characters had an atypical allele succession respect to the founder or the most frequent STs of the respective CC. In brackets the number of isolates.

**Table 2:** Relatedness between ribogroups, *spa* types, *spa* CCs, and clusters as per BURP analysis.

Ribogroup	MLST CC	MLST	*spa* Type	*spa* CC	Cluster
Ribocluster (48)	CC30 (48)	ST30 (31)	t012 (12) t021 (8) t018 (3) t093 (1) t338 (3) t1382 (2) t5891 (1) t11956 (1)	**CC021/012** (37)	**Cluster 1**
		ST2954 ** (3)	t298 (2) t012 (1)		
		ST2957 ** (1) ST2960 ** (1) ST untyp. (1)	t021 (1) t021 (1) t338 (1)		
		ST34 (8)	t166 (4) t4437 (1) t369 (3)	**CC166** (8)	**Cluster 4**
			t3906 (1)	**Singleton** (1)	-
		ST34 (1) ST30 (1)	t13129 * (1) t13132 * (1)	***spa*types with missing alignments** (2)	-
*cra*-94-S-7 (16)	CC5 (15)	ST228 (7) ST228 (6) ST2956 ** (1)	t001 (7) t041 (6) t109 (1)	**CC002** (14)	**Cluster 2**
		ST228 (1)	t1382 (1)	**CC021/012** (1)	**Cluster 1**
	CC45 (1)	ST46 (1)	t1646 (1)	**Singleton** (1)	-
*cra*-146-S-8 (9)	CC5 (9)	ST5 (8)	t002 (2) t179 (1) t548 (2) t985 (1)	**CC002** (6)	**Cluster 2**
			t045 (1)	**Singleton** (2)	-
			t5349 (1)		
		ST2616 (1)	t13130 * (1)	***spa*type with missing alignments** (1)	-
*cra*-137-S-4 (14)	CC8 (14)	ST8 (9)	t008 (5) t024 (2) t121 (1) t574 (1)	**CC121** (13)	**Cluster 3**
		ST247 (4)	t051 (4)		
		ST241 (1)	t037 ^†^(1)	**CC021/012** (1)	**Cluster 1**
*cra*-157-S-4 (13)	CC7 (10)	ST7 (9) ST2955 ** (1)	t091 (9) t091 (1)	**Singleton** (13)	-
	CC1 (2)	ST109 (2)	t209 (2)		
	CC n.d.	ST101 (1)	t7956 (1)		
*cra*-147-S-6 (8)	CC1	ST1 (8)	t127 (5) t2478 (1)	**Singleton** (6)	-
			t693 (1) t1252 (1)	**Excluded CC no founder** (2)	**Cluster 5**

The underlined strains t1382 belonged to different ribogroups and exhibit different STs, with diverse allelic profiles (ST30 and ST228); †: *spa* type t037 belonging to CC021/012 was found in a different ribogroup and MLST CC; t693 and t1252 were excluded due to the shorter repetitions (<5): t693 (7), t1252 (07-22-13); * new *spa* types found in this study and submitted to the *spa* Ridom software; ** new STs founded in this study and to be submitted to the MLST database. In brackets are the number of isolates.

**Table 4:** Atypical STs and *spa* types in relation with the most frequent genetic profile observed in the same ribogroup.

Ribogroup	STs	MLST Allele Succession (*arc-aroe-glpf-gmk-pta-tpi-yiql*)	*agr* Type	*spa* Types-*spa* CC	*spa* Repeats Succession
*cra*-94-S-7 (16)	ST228 (14) **ST46** (1)	01-04-01-04-12-24-29 *10–14-08–06-14-03-02*	II I	t001-CC002 (7) t041-CC002 (6) **t1646**–singleton (1)	26-30-17-34-17-20-17-12-17-16 26-30-17-34-17-20-17-34-17-20-17-12-17-16 *09-20-16-34-13-17-34-16-34*
*cra*-157-S-4 (13)	ST7 (9) **ST109** (2) **ST101** (1)	05-04–01-04-04-06-03 *03-27-01-01–01-01-1003-01-14-15-11-19-03*	I II I	t091-singleton (9) **t209**-singleton (2) **t7956**-singleton (1)	07-23-21-17-34-12-23-02-12-23 *07-16-12-23-3404-13-21-12-17-20-17-12-17-17-17*
*cra*-137-S-4 (14)	ST8 (9) ST241 (1)	03-03-01-01-04–04–03 02-03-01-01-04–04–03	I I	t008 (5 CC121) **t037** (1 CC021/012)	11-19-12-21-17-34-24-34-22-25 *15-12-16-02-25-17-24*

STs and *spa* types atypical for the ribogroup appear in bold. MLST allelic profile and *spa* repeats successions that are unusual within the ribogroup are in italics. In brackets the number of isolates.

**Table 5:** Hunter–Gaston discriminatory index of *spa* typing and MLST of 108 *S. aureus*.

	*Spa* Typing	MLST	Ribotyping	*spa* CC	MLST CC
MSSA (79)	0.936	0.802 *	0.596	0.746 ^†^	0.590 **
MRSA (29)	0.879	0.751	0.636	0.594	0.567
TOTAL (108)	0.955	0.869 *	0.744	0.817 ^†^	0.721 **

MSSA methicillin-susceptible *S. aureus*, MRSA methicillin-resistant *S. aureus*, * Excluding one non typeable ST (Total n° = 107, MSSA n° = 78), ** Excluding one strain without CC (Total n° = 107, MSSA n°=78). **^†^** Excluding two strains with no founder CC (due to the shorter repetitions i.e., <5). In brackets the number of isolates.

**Table 6:** Clonal Complexes and relationship between the genotypic patterns of the 79-methicillin susceptible *S. aureus* (MSSA).

Ribogroup ^1^	MLST CC ^1^	MLST ST	*spa* Type	*spa* CC	IS*256*
Ribocluster (48/48)	CC30 (48/48)	**ST30** (31), **ST34** (10), ST2954 (3), ST2957 (1), ST2960 (1), ST243 (1), ST non-typeable (1)	**t012** (13), **t021** (10), t018 (3), t093 (1), t166 (4), t298 (2), t338 (4), t369 (3), t1382 (2), t3906(1), t4437 (1), t5891 (1), t11956 (1), t13129 * (1), t13132 * (1)	**CC021/012** (37) CC166 (8) singleton and missing alignments (3)	neg (44)
*cra*-94-S7 (2/16) *cra*-146-S8 (7/9)	CC5 (8/25) CC45 (1/25)	**ST5** (6), ST228 (1), ST2626 (1) ST46 (1)	t1382 (1), t1646 (1), t002 (1), t045 (1), t179(1), t548 (2), t5349 (1), t13130 * (1)	**CC002** (5) CC021/012 (1) singletons (3)	neg (8) pos (1)
*cra*-137-S4 (3/14)	CC8 (3/14)	**ST8** (3)	t008 (1), t024 (1), t574 (1)	**CC121** (3)	neg (3)
*cra*-157-S4 (13/13)	CC7 (10/13) CC1 (2/13) CC not delivered (1/13)	**ST7** (9)–ST2955 (1) ST109 (2) ST101 (1)	**t091** (10), t209 (2), t7956 (1)	singletons (13)	neg (13)
*cra*-147-S6 (6/8)	CC1 (6/8)	**ST1** (6)	**t127** (3), t693 (1), t1252 (1), t2478 (1)	excluded (2) singletons (4)	neg (4) pos (2)

Numbers between brackets represent the number of strains. The Clonal Complexes of STs and the Clonal Complexes of *spa* types were inferred by eBURST v3 and BURP analysis, respectively, on the entire collection of 108 *S. aureus*. The main ST type, *spa* type, and *spa* CC appear in bold. ^1^ (number of MSSA isolates /number of isolates of the ribogroup). * Excluding one non typeable ST (Total n° = 107, MSSA n° = 78).

**Table 7:** Clonal Complexes and relationship between the genotypic patterns of the 29 methicillin resistant *S. aureus* (MRSA).

Ribogroup ^1^	MLST CC ^1^	MLST ST	*spa* Type	*spa* CC	IS*256*
*cra*-94-S7 (14/16) *cra*-146-S8 (2/9)	CC5 (16/25)	**ST228** (13) ST5 (2) ST2956 (1)	**t001** (7), **t041** (6), t109 (1), t002 (1), t985 (1)	**CC002** (16)	pos (14) neg (2)
*cra*-137-S4 (11/14)	CC8 (11/14)	**ST8** (6) ST247 (4) ST241 (1)	**t008** (4), **t051** (4), t024 (1), t037 (1), t121 (1)	**CC121** (10) singleton (1)	pos (7) neg (4)
*cra*-147-S6 (2/8)	CC1 (2/8)	**ST1** (2)	**t127** (2)	singletons (2)	pos (2)

Numbers between brackets represent the number of strains. The Clonal Complexes of STs and the Clonal Complexes of *spa* types were inferred by eBURST v3 and BURP analysis respectively on the entire collection of 108 *S. aureus*. The main ST type, *spa* type, and *spa* CC appear in bold. ^1^ (number of MSSA isolates /number of isolates of the ribogroup).

## Data Availability

The data presented in this study are available on request from the corresponding authors (C.R.A. and D.C.).
